# Quantitative analysis of the impact of respiratory state on the heartbeat-induced movements of the heart and its substructures

**DOI:** 10.1186/s13014-023-02396-0

**Published:** 2024-02-05

**Authors:** Benzhe Liang, Guanzhong Gong, Ying Tong, Lizhen Wang, Ya Su, Huadong Wang, Zhenkai Li, Hongyu Yan, Xiaohong Zhang, Yong Yin

**Affiliations:** 1 College of materials science and technology, Nanjing University of Aeronautics and Astronautics, Nanjing, China; 2 Department of Radiation Oncology Physics and Technology, Shandong Cancer Hospital and Institute, Shandong First Medical University, Shandong Academy of Medical Sciences, Jinan, China

**Keywords:** DIBH, EIBH, EEBH, Heart and its substructures, Displacement, Volume, DSC

## Abstract

**Purpose:**

This study seeks to examine the influence of the heartbeat on the position, volume, and shape of the heart and its substructures during various breathing states. The findings of this study will serve as a valuable reference for dose-volume evaluation of the heart and its substructures in radiotherapy for treating thoracic tumors.

**Methods:**

Twenty-three healthy volunteers were enrolled in this study, and cine four-dimensional magnetic resonance images were acquired during periods of end-inspiration breath holding (EIBH), end-expiration breath holding (EEBH), and deep end-inspiration breath holding (DIBH). The MR images were used to delineate the heart and its substructures, including the heart, pericardium, left ventricle (LV), left ventricular myocardium, right ventricle (RV), right ventricular myocardium (RVM), ventricular septum (VS), atrial septum (AS), proximal and middle portions of the left anterior descending branch (pmLAD), and proximal portion of the left circumflex coronary branch (pLCX). The changes in each structure with heartbeat were compared among different respiratory states.

**Results:**

Compared with EIBH, EEBH increased the volume of the heart and its substructures by 0.25–3.66%, while the average Dice similarity coefficient (DSC) increased by − 0.25 to 8.7%; however, the differences were not statistically significant. Conversely, the VS decreased by 0.89 mm in the left–right (LR) direction, and the displacement of the RV in the anterior–posterior (AP) direction significantly decreased by 0.76 mm (*p* < 0.05). Compared with EIBH and EEBH, the average volume of the heart and its substructures decreased by 3.08–17.57% and 4.09–20.43%, respectively, during DIBH. Accordingly, statistically significant differences (*p* < 0.05) were observed in the volume of the heart, pericardium, LV, RV, RVM, and AS. The average DSC increased by 0–37.04% and − 2.6 to 32.14%, respectively, with statistically significant differences (*p* < 0.05) found in the right ventricular myocardium and interatrial septum. Furthermore, the displacements under DIBH decreased in the three directions (i.e.,− 1.73 to 3.47 mm and − 0.36 to 2.51 mm). In this regard, the AP displacement of the heart, LV, RV, RVM, LR direction, LV, RV, and AS showed statistically significant differences (*p* < 0.05). The Hausdorff distance (HD) of the heart and its substructures under the three breathing states are all greater than 11 mm.

**Conclusion:**

The variations in the displacement and shape alterations of the heart and its substructures during cardiac motion under various respiratory states are significant. When assessing the dose-volume index of the heart and its substructures during radiotherapy for thoracic tumors, it is essential to account for the combined impacts of cardiac motion and respiration.

## Introduction

The thoracic region is prone to developing malignancies such as lung, esophageal, and breast cancer, which can be treated with radiotherapy [1–3]. However, this poses a significant risk to patient survival time and quality of life, with radiation-induced heart damage being one of the most common complications [4–7]. Numerous studies have suggested that radiation-induced heart damage might offset the clinical benefits of radiotherapy, underlining the importance of early detection [8]. Current clinical methodology predicts such damage using dose-volume indices of the heart obtained from static simulation positioning 3DCT, which ignores the effects of breathing and cardiac motion on the heart's position, volume, and shape. Studies have indicated that the dose-volume indices differ significantly from the actual dose [9]. In past research, when conducting electrocardiogram-gated 4DCT scans under end-inspiration breath holding (EIBH), we observed significant cardiac shape and volume changes throughout the cycle, strongly affecting dosage evaluation accuracy [10]. When a patient's breathing state shifts, it changes the thoracic pressure, affecting the heart's range of motion and shape modulation. Unfortunately, few trials have sought to analyze the changes in the position, volume, and shape of the heart and its substructures under various respiratory states. Our study aims to examine these changes in three breathing conditions: EIBH, tidal end-expiratory breath holding (EEBH) and deep end-inspiration breath holding (DIBH).

## Materials and methods

### General study object information

The study included 23 participants, aged between 20 and 28 years, with a median age of 23 years, comprising 12 males and 11 females, all of whom demonstrated a breath-holding capacity of over 20 s during a single breath-hold in EIBH, EEBH and DIBH. Prior to participating in the study, all volunteers gave their informed consent, and the study was approved by the Ethics Committee of Shandong Cancer Hospital.

### Acquisition of 4D MR images

All study participants underwent thoracic imaging with a GE 3.0 T superconducting MR scanner (Discovery 750 W, General Electric, USA) while maintaining EIBH, EEBH and DIBH, in which electrocardiogram gating was utilized to obtain 4D MR images. The scanning sequence used was a multislice 2D fast imaging employing steady-state acquisition (FIESTA). Scans had a slice thickness of 5 mm and a field of view measuring 50 cm and were performed without a gap. 4D MR images were reconstructed over 20 phases, ranging from 0 to 95% in 5% intervals, synchronized with the cardiac cycle.

### Outlining of the heart and its substructures

Using MIM Maestro 7.1.5 (MIM, USA), the 4D MR images were manually segmented for different respiratory phases. In each image phase, the heart, pericardium, ventricular septum (VS), left and right ventricular myocardium (LVM and RVM, respectively), left ventricle (LV), right ventricle (RV), atrial septum (AS), proximal and middle portions of the left anterior descending coronary branch (pmLAD), and the proximal portion of the left circumflex coronary branch (pLCX) were segmented.

The upper boundary of the heart and pericardium is defined as the roof of the left atrium, and the lower boundary is the apex of the heart. The upper boundary of the left and right ventricles is the top of their respective chambers, and the lower boundary is the bottom of their chambers. The upper and lower boundaries of the left and right ventricular myocardium are the same as those of their corresponding chambers. The upper and lower boundaries of the interventricular septum are the same as those of the left ventricular myocardium. The upper boundary of the interatrial septum is the roof of the right atrium, and the lower boundary is the junction of the left atrium and left ventricle. The left anterior descending branch of the coronary artery starts at the bifurcation of the left main stem and ends at the bifurcation of the second diagonal branch. The left circumflex artery begins at the bifurcation of the left main stem and ends at the midpoint between the left margin of the heart and the posterior interventricular groove. All delineations were performed by two physicians jointly, and in case of any doubts, a third physician was involved in the discussion to determine the delineations.

### Comparative analysis

We compared the displacement in the center of mass of the heart and its substructures in the left–right (LR), anterior–posterior (AP), and cranio-caudal (CC) directions during the cardiac cycle across the different respiratory states. Additionally, we compared the morphological and volumetric changes in the heart and its substructures. We calculated the volumetric variations by dividing the difference between the maximum and minimum volumes by the minimum volume. We evaluated the change in shape using the dice similarity coefficient (DSC).

$${\text{DSC}} = \frac{{2\left| {A \cap B} \right|}}{\left| A \right| + \left| B \right|}$$

here A refers to the volume of the region of interest (ROI) at the reference phase (0%), while B represents the volume of the ROI at other phases. The overlapping volume between the two is represented as |A ∩ B|.

The Hausdorff distance (HD) is a measure of the similarity between two sets of points. It is a definition of the distance between two point sets: Suppose there are two sets A = {a1,…, ap}, B = {b1,…,bq}; then, the HD between these two point sets is defined as:

$$HA,B = {\text{max}}\left( {\underbrace {{{\text{max}}}}_{a \in A}\left\{ {\underbrace {\min }_{b \in B}\left\| {a - b} \right\|} \right\},\underbrace {{{\text{max}}}}_{b \in B}\left\{ {\underbrace {{{\text{min}}}}_{a \in A}\left\| {b - a} \right\|} \right\}} \right)$$

HD is measured in millimeters (mm), and the higher the HD is, the greater the difference.

### Statistical methods

SPSS 25 (IBM, Chicago, USA) software was used to analyze the data, and the results are presented as the mean ± standard deviation (${\overline{\text{x}}}$  ± s). We applied paired t tests to compare normally distributed data and Mann‒Whitney U tests to compare nonnormally distributed data. Significance was indicated if *p* < 0.05.

## Results

### Analysis of the movement of and morphological changes in the heart and its substructures during EIBH at end-expiration

**Fig. 1:**
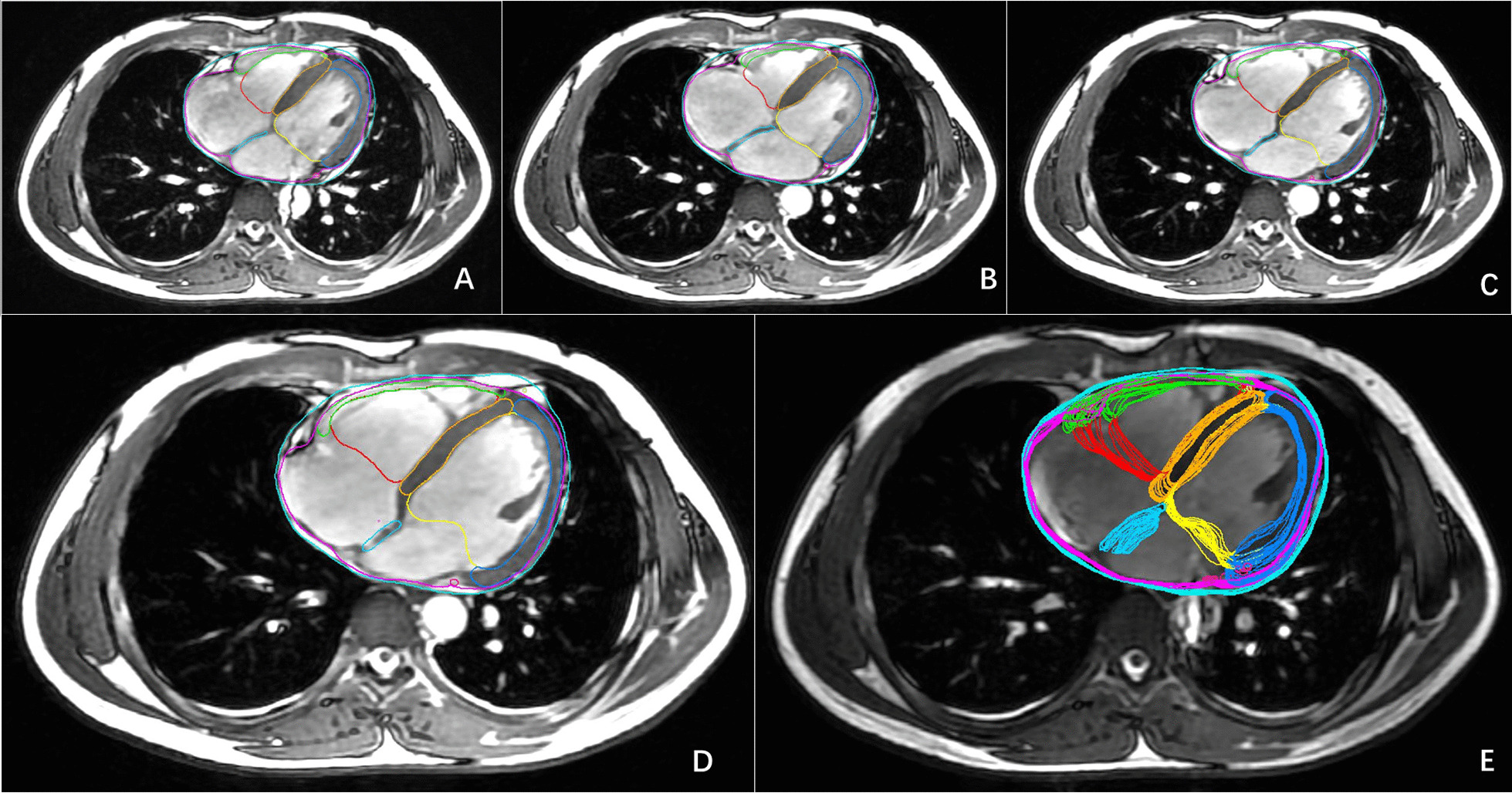
Changes in the position and shape of the heart and its substructures at the end of a calm inhalation at the same level. The positions and shapes of the heart and its substructures are shown in the following time phases: **A** 0% phase, **B** 25% phase, **C** 50% phase, **D** 75% phase, and **E** the overall display of the heart and its substructures over the 20 time phases

During EIBH at end-expiration, the heart and its substructures were displaced by an average of 1.4–10.73 mm, 0.68–12.57 mm, and 2.31–13.18 mm in the LR, AP, and CC directions, respectively (Table 1). The volume variations ranged from 13.45 to 136.53% (Table 2), the DSC variations ranged from 6.75 to 2318.06% (Table 3), and the HD_MAX_ ranged from 11.8 to 27.28 mm (Table 4). The RVM exhibited the greatest displacement in the left–right direction (10.73 mm), while the pmLAD showed the greatest displacement in the AP and CC directions (12.57 mm and 13.18 mm, respectively), and the pericardium demonstrated the smallest displacement in all directions (1.4 mm, 0.68 mm, and 2.31 mm, respectively). The LV displayed the largest volume variations (136.53%), and the pericardium displayed the smallest (13.45%). The AS DSC exhibited the greatest change (2318.06%), and the pericardium DSC exhibited the least (6.75%). Statistically significant differences (*p* < 0.05) were observed between the maximum and minimum values of each structure's volume and DSCs (Tables 2 and 3). The LVM displayed the largest HDMAX value (27.28 mm), and the pericardium displayed the smallest HD_MAX_ value (11.8 mm) (Fig. 1).

**Table 1:** Comparison of the displacement of the centroid of the heart and its substructures under three respiratory states

	LR	AP	CC
*HEART*			
EIBH	1.86 ± 0.60	1.03 ± 0.33	2.81 ± 1.17
EEBH	1.79 ± 0.58	1.07 ± 0.38	3.31 ± 0.77
DIBH	1.38 ± 0.45	1.02 ± 0.54	3.08 ± 1.14
*Pericardium*			
EIBH	1.19 ± 0.54	0.64 ± 0.28	2.21 ± 1.05
EEBH	1.40 ± 0.51	0.68 ± 0.28	2.31 ± 0.89
DIBH	0.94 ± 0.42	0.86 ± 0.52	2.26 ± 0.87
*LV*			
EIBH	2.99 ± 1.01	4.61 ± 1.02	3.41 ± 1.00
EEBH	3.11 ± 0.66	4.73 ± 0.92	3.38 ± 1.13
DIBH	2.36 ± 1.41	4.08 ± 1.00	3.41 ± 1.40
*LVM*			
EIBH	2.63 ± 1.16	7.1 ± 2.32	4.15 ± 1.17
EEBH	2.91 ± 0.98	7.12 ± 2.00	4.16 ± 1.35
DIBH	2.79 ± 0.87	6.77 ± 2.11	3.74 ± 1.35
*RV*			
EIBH	7.96 ± 2.41	4.68 ± 1.22	4.67 ± 1.28
EEBH	6.97 ± 2.67	3.92 ± 1.36	5.04 ± 1.93
DIBH	5.87 ± 1.70	3.76 ± 1.36	4.60 ± 1.73
*RVM*			
EIBH	11.69 ± 3.42	7.65 ± 2.63	6.02 ± 1.49
EEBH	10.73 ± 3.5	6.97 ± 2.12	5.98 ± 2.80
DIBH	8.22 ± 3.30	5.09 ± 1.76	5.62 ± 1.67
*VS*			
EIBH	5.50 ± 1.75	4.28 ± 0.96	4.13 ± 1.62
EEBH	4.61 ± 1.59	4.16 ± 1.04	4.76 ± 1.73
DIBH	3.52 ± 1.70	3.62 ± 1.22	4.54 ± 1.40
*AS*			
EIBH	3.56 ± 1.14	6.43 ± 1.61	6.42 ± 2.28
EEBH	3.29 ± 1.05	6.87 ± 1.51	6.63 ± 2.63
DIBH	3.03 ± 1.08	4.82 ± 1.96	6.12 ± 2.15
*pmLAD*			
EIBH	5.44 ± 3.72	11.53 ± 6.28	11.81 ± 2.26
EEBH	5.37 ± 1.86	12.57 ± 6.24	13.18 ± 3.95
DIBH	4.42 ± 2.00	11.72 ± 5.36	13.54 ± 5.16
*pLCX*			
EIBH	8.29 ± 3.73	9.43 ± 3.60	14.38 ± 4.65
EEBH	7.61 ± 3.54	8.52 ± 3.21	11.86 ± 4.31
DIBH	5.75 ± 2.13	8.00 ± 3.91	11.13 ± 3.56

*LR* left–right, *AP* anterior–posterior, *CC* cranio-caudal

**Table 2:** Comparison of volume changes in the heart and its substructures during the three respiratory states (X ± S, mm)

	volume_MAX_	volume_MIN_	volume_AVE_	Variation	*p* value
*HEART*					
EIBH	609.34 ± 141.9	519.2 ± 128.24	570.84 ± 137.94	17.82 ± 3.79	0.000
EEBH	607.26 ± 143.63	525.96 ± 136.48	573.48 ± 140.23	15.98 ± 4.4	0.000
DIBH	529.69 ± 134.56	451.4 ± 119.76	493.89 ± 130.25	17.79 ± 5.04	0.000
*Pericardium*					
EIBH	692.53 ± 157.69	612.18 ± 145.38	659.02 ± 154.72	13.45 ± 3.33	0.000
EEBH	691.43 ± 156.56	615.47 ± 149.94	660.71 ± 154.01	12.74 ± 3.8	0.000
DIBH	607.72 ± 147.75	533.91 ± 132.92	574.02 ± 142.94	14.07 ± 4.25	0.000
*LV*					
EIBH	151.35 ± 33.25	66.5 ± 22.81	113.33 ± 28.7	136.53 ± 33.41	0.000
EEBH	150.45 ± 35.63	71.62 ± 28.31	114.47 ± 31.81	120.12 ± 32.54	0.000
DIBH	134.36 ± 37.68	62.2 ± 26.6	100.88 ± 31.36	131.31 ± 51.1	0.000
*LVM*					
EIBH	52.49 ± 11.19	41.22 ± 10	47.1 ± 10.49	28.14 ± 8.39	0.000
EEBH	52.01 ± 12.53	42.13 ± 11.1	47.6 ± 11.2	24.71 ± 10.72	0.000
DIBH	50.42 ± 10.96	41.04 ± 9.83	45.65 ± 10.05	24.08 ± 11.11	0.000
*RV*					
EIBH	105.88 ± 25.94	49.51 ± 16.39	79.46 ± 21.98	122.37 ± 36.67	0.000
EEBH	108.61 ± 25	50.35 ± 17.25	82.37 ± 22.84	123.75 ± 32.67	0.000
DIBH	94.37 ± 26.67	46.76 ± 16.59	72.38 ± 22.79	109.43 ± 41.2	0.000
*RVM*					
EIBH	34.73 ± 9.58	18.13 ± 5.67	26.66 ± 7.14	99.54 ± 54.04	0.000
EEBH	35.23 ± 9.06	18.37 ± 4.86	27.36 ± 7.41	95 ± 28.39	0.000
DIBH	30.23 ± 6.11	18.87 ± 5.61	24.36 ± 5.61	66.91 ± 31.22	0.000
*VS*					
EIBH	32.61 ± 8.35	27.67 ± 8.28	30.08 ± 8.23	19.24 ± 9.23	0.000
EEBH	32.93 ± 8.43	27.89 ± 7.43	30.35 ± 7.83	18.38 ± 5.08	0.000
DIBH	32.44 ± 8.95	26.27 ± 7.09	29.03 ± 7.57	23.5 ± 8.08	0.000
*AS*					
EIBH	2.94 ± 1.07	1.63 ± 0.78	2.22 ± 0.89	94.86 ± 59.85	0.000
EEBH	3.17 ± 1.05	1.6 ± 0.66	2.3 ± 0.77	108.61 ± 42.51	0.000
DIBH	2.41 ± 0.69	1.35 ± 0.48	1.83 ± 0.55	89.26 ± 42.75	0.000

*volume*_*MAX*_ maximum volume, *volume*_*MIN*_ minimum volume, *volume*_*AVE*_ average volume, *variation* volume variations

**Table 3:** Comparison of Dice similarity coefficient (DSC) changes in the heart and its substructures during the three respiratory states. (${\overline{\text{X}}}$ ± S, mm)

	DSC_MAX_	DSC_MIN_	DSC_AVE_	Variation	*p* value
*HEART*					
EIBH	0.99 ± 0.01	0.9 ± 0.01	0.94 ± 0.01	9.44 ± 2.13	0.000
EEBH	0.99 ± 0.004	0.9 ± 0.02	0.93 ± 0.01	9.97 ± 1.93	0.000
DIBH	0.99 ± 0.01	0.9 ± 0.02	0.94 ± 0.01	9.66 ± 2.14	0.000
*Pericardium*					
EIBH	0.99 ± 0.01	0.93 ± 0.01	0.95 ± 0.01	6.75 ± 1.39	0.000
EEBH	0.995 ± 0.003	0.93 ± 0.02	0.95 ± 0.01	6.9 ± 1.95	0.000
DIBH	0.99 ± 0.004	0.93 ± 0.02	0.96 ± 0.01	6.82 ± 1.73	0.000
*LV*					
EIBH	0.98 ± 0.01	0.62 ± 0.06	0.75 ± 0.05	57.61 ± 9.67	0.000
EEBH	0.98 ± 0.01	0.65 ± 0.07	0.77 ± 0.05	51.73 ± 10.68	0.000
DIBH	0.98 ± 0.02	0.62 ± 0.09	0.75 ± 0.06	57.81 ± 14.79	0.000
*LVM*					
EIBH	0.97 ± 0.01	0.2 ± 0.08	0.46 ± 0.07	393.47 ± 41.61	0.000
EEBH	0.97 ± 0.01	0.26 ± 0.1	0.5 ± 0.07	276.3 ± 39.71	0.000
DIBH	0.96 ± 0.04	0.24 ± 0.13	0.51 ± 0.08	299 ± 60.54	0.000
*RV*					
EIBH	0.98 ± 0.01	0.61 ± 0.06	0.75 ± 0.04	59.65 ± 9.88	0.000
EEBH	0.98 ± 0.01	0.60 ± 0.06	0.75 ± 0.04	62.05 ± 10.16	0.000
DIBH	0.98 ± 0.01	0.64 ± 0.09	0.77 ± 0.05	52.03 ± 13.72	0.000
*RVM*					
EIBH	0.93 ± 0.04	0.14 ± 0.05	0.4 ± 0.03	574.85 ± 47.93	0.000
EEBH	0.95 ± 0.02	0.12 ± 0.06	0.39 ± 0.05	670.33 ± 49.85	0.000
DIBH	0.93 ± 0.04	0.2 ± 0.12	0.44 ± 0.08	369.52 ± 72.76	0.000
*VS*					
EIBH	0.96 ± 0.01	0.53 ± 0.09	0.7 ± 0.05	82.24 ± 17.48	0.000
EEBH	0.96 ± 0.02	0.52 ± 0.08	0.71 ± 0.05	83.72 ± 13.94	0.000
DIBH	0.96 ± 0.02	0.57 ± 0.09	0.71 ± 0.05	69.53 ± 17.29	0.000
*AS*					
EIBH	0.86 ± 0.06	0.04 ± 0.05	0.27 ± 0.09	2318.06 ± 171.31	0.000
EEBH	0.85 ± 0.07	0.04 ± 0.06	0.28 ± 0.09	1889.85 ± 177.15	0.000
DIBH	0.88 ± 0.08	0.11 ± 0.09	0.37 ± 0.09	736.97 ± 124.34	0.000

*DSC* dice similarity coefficient, *DSC*_*MAX*_ maximum DSC, *DSC*_*MIN*_ minimum DSC, *DSC*_*AVE*_ average DSC, *Variation* DSC variations

**Table 4:** Comparison of Hausdorff distance (HD) of the heart and its substructures under three respiratory states. (${\overline{\text{X}}}$ ± S, mm)

		HD_MAX_	HD_AVE_			HD_MAX_	HD_AVE_
HEART	EIBH	15.2 ± 2.28	11.21 ± 1.31	RVM	EIBH	27.28 ± 12.86	19.05 ± 12.01
	EEBH	15.54 ± 2.52	11.39 ± 1.54		EEBH	24.32 ± 3.84	15.92 ± 2.56
	DIBH	14.66 ± 2.19	11.07 ± 1.59		DIBH	21.91 ± 5.49	15.19 ± 3.31
Pericardium	EIBH	11.8 ± 2.71	8.84 ± 1.81	VS	EIBH	17.41 ± 4.08	11.56 ± 2.5
	EEBH	12.08 ± 2.76	9.13 ± 2.02		EEBH	15.6 ± 3.05	10.15 ± 1.67
	DIBH	12.96 ± 3.07	9.67 ± 2.56		DIBH	15.22 ± 3.94	10.57 ± 2.08
LV	EIBH	17.69 ± 2.79	12.39 ± 1.99	AS	EIBH	13.83 ± 3.18	8.96 ± 2.11
	EEBH	16.05 ± 2.49	11.2 ± 1.44		EEBH	13.7 ± 3.99	9.03 ± 2.63
	DIBH	17.75 ± 2.85	12.73 ± 1.78		DIBH	11.53 ± 2.3	8 ± 1.84
LVM	EIBH	19.5 ± 4.23	13.78 ± 2.86	pmLAD	EIBH	19.79 ± 8.25	12.84 ± 5.96
	EEBH	19.52 ± 3.54	13.5 ± 2.5		EEBH	17.68 ± 8.04	10.55 ± 3.27
	DIBH	18.49 ± 3.8	13.38 ± 2.27		DIBH	17.51 ± 7.38	11.75 ± 6.61
RV	EIBH	20.24 ± 2.27	13.41 ± 1.57	pLCX	EIBH	18.98 ± 4.12	12.42 ± 2.96
	EEBH	21.23 ± 3.71	13.9 ± 2.59		EEBH	21.26 ± 5.51	14.12 ± 4.01
	DIBH	19.47 ± 4.56	13.3 ± 2.6		DIBH	18.63 ± 5.27	12.35 ± 3.58

*HD*_*MAX*_ maximum HD, *HD*_*AVE*_ average volume

### Analysis of the movement and morphological changes of the heart and its substructures during EEBH at end-expiration

During EEBH at end-expiration, the heart and its substructures showed a mean displacement range of 1.19–11.69 mm, 0.64–11.53 mm, and 2.21–14.38 mm in the LR, AP, and CC directions, respectively (Table 1). The volume variations ranged from 12.74 to 123.75% (Table 2), and the DSC variations ranged from 6.9 to 1889.85% (Table 3). Except for pLCX, which exhibited the largest CC displacement at 14.38 mm, the RV volume variations were the greatest at 123.75%, which was different from the results of EIBH at end-expiration. The other structures remained unchanged regarding maximum and minimum displacement, volume variations, and DSC variations relative to the results of EIBH at end-expiration. Statistically significant differences (*p* < 0.05) were observed between the maximum and minimum values of each structure's volume and DSC (Tables 2 and 3). The LVM displayed the largest HDMAX value (24.32 mm), and the pericardium displayed the smallest HDMAX value (12.08 mm).

**Fig. 2:**
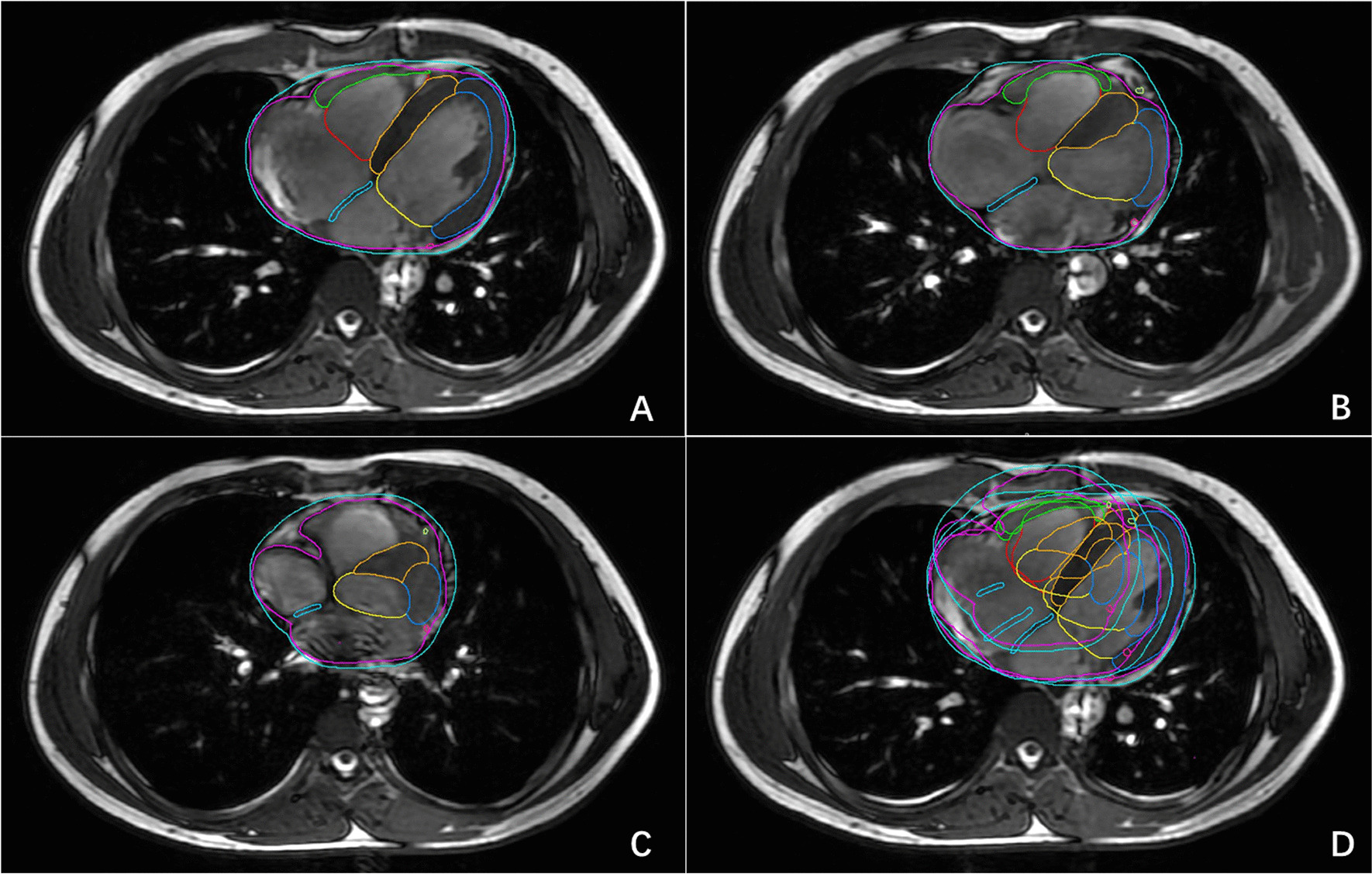
Comparison of the position and shape of the heart and its substructures at the same level during the three respiratory states at the 0% phase. **A** Position and shape of the heart and its substructures at the end of calm exhalation, **B** position and shape of the heart and its substructures at the end of calm inhalation, **C** position and shape of the heart and its substructures at the end of deep inhalation, and **D** display of the position and shape of the heart and its substructures under the three respiratory states on an image obtained at the end of calm exhalation

Compared to EIBH at end-expiration, the volume of the heart and its substructures increased by 0.25–3.66% under EEBH at end-expiration, but the differences were not statistically significant. The displacement of the heart and its substructures decreased in the LR direction by − 0.28 to 0.99 mm, in the AP direction by − 1.04 to 0.91 mm, and in the CC direction by − 1.37 to 2.52 mm. Only the displacement of the VS in the LR direction and the RV in the AP direction exhibited statistically significant differences (*p* < 0.05). The average DSC of the heart and its substructures increased by − 2.5 to 8.7%, but the differences were not statistically significant. HD_MAX_ has been reduced to a range of − 2.28 to 2.96 mm, with the maximum reduction being in the RVM (Fig. 2).

### Analysis of the movement and morphological changes of the heart and its substructures during DIBH at end-inhalation

During DIBH at end-inhalation, the heart and its substructures were displaced by an average of 0.94–8.22 mm, 0.86–11.72 mm, and 2.26–13.54 mm in the LR, AP, and CC directions, respectively (Table 1). The volume variations ranged from 14.07 to 131.31% (Table 2), the DSC variations ranged from 6.82 to 736.97% (Table 3), and the HD_MAX_ ranged from 11.53 to 21.91 mm (Table 4). The structures with the largest and smallest displacement, volume variations, and DSC variations remained unchanged from the results of EIBH at end-inhalation. Statistically significant differences (*p* < 0.05) were observed between the maximum and minimum values of each structure's volume and DSC (Tables 2 and 3). The RVM displayed the largest HDMAX value (21.91 mm), and the AS displayed the smallest HDMAX value (11.53 mm).

Compared to EIBH at end-inhalation, the volume of the heart and its substructures decreased by 3.08–17.57% during DIBH at end-inhalation. Statistically significant differences (*p* < 0.05) were observed in the heart, pericardium, LV, RV, RVM, and AS but not in the LVM and VS. Displacements in the LR direction decreased by − 0.16 to 3.47 mm, in the AP direction by − 0.22 to 2.56 mm, and in the CC direction by − 1.73 to 3.25 mm. Statistically significant differences (*p* < 0.05) were observed in the LR direction of the heart, pericardium, LV, RV, RVM, and VS and in the AP direction of the LV, RVM, and AS. The average DSC of the heart and its substructures increased by 0–37.04%, with statistically significant differences (*p* < 0.05) for the LVM, RVM, and AS, but not for the heart, pericardium, LV, RV, or VS. HD_MAX_ was reduced to a range of − 1.16 to 5.37 mm, with the maximum reduction being in the RVM (Fig. 2).

Relative to those during EEBH at end-expiration, the volume of the heart and its substructures decreased by 4.09–20.43% during DIBH at end-inhalation, with statistically significant differences (*p* < 0.05) in the heart, pericardium, LV, RV, RVM, VS, and AS, but not in LVM. Displacements in the LR direction decreased by 0.12–2.51 mm, in the AP direction by − 0.18 to 2.05 mm, and in the CC direction by − 0.36 to 0.73 mm. Statistically significant differences (*p* < 0.05) were observed in the LR direction of the heart, LV, RV, RVM, and VS, as well as in the AP direction of LV, RV, RVM, AS, and pLCX. In the CC direction, only pLCX exhibited statistically significant differences. The average DSC of the heart and its substructures increased by − 2.6 to 32.14%, with statistically significant differences (*p* < 0.05) observed only in the RVM and AS. HD_MAX_ has been reduced to a range of − 1.7 to 2.63 mm, with the maximum reduction being in the pLCX.

**Fig. 3:**
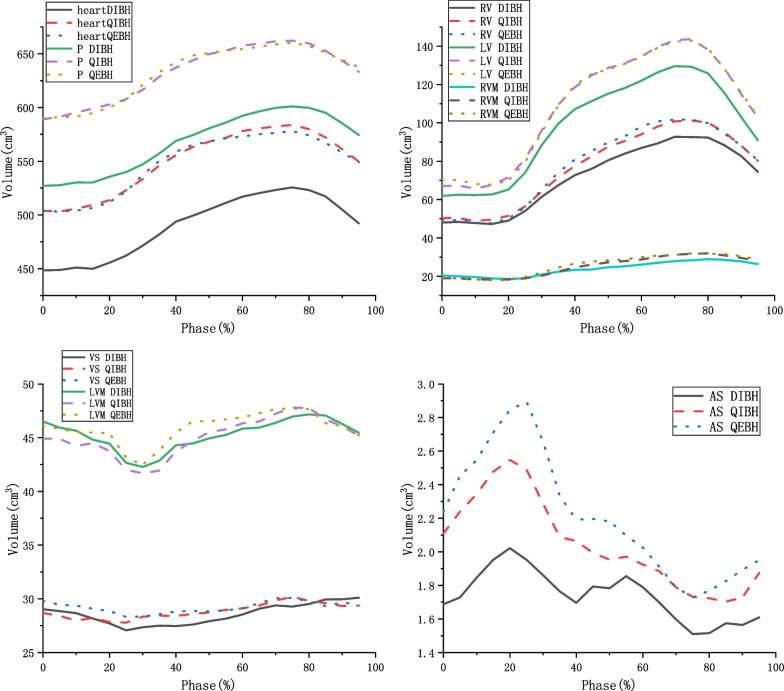
Volume changes in the heart and its substructures at different time phases during the three respiratory states

Figure 3 illustrates the volume changes of the heart and its substructures at different time points. There was good consistency in the volume changes in the heart, pericardium, LV, RV, and RVM. Additionally, there was good consistency in the volume changes in the LVM and the AS. Among the three breathing states, the volume of the heart and its substructures was at its lowest during DIBH.

## Discussion

This study is the only quantitative analysis of the changes in the position, volume, and shape of the heart and its substructures during three different breathing states, EIBH, EEBH, and DIBH, over the cardiac cycle.

Radiation-induced cardiac injury is a leading cause of nontumor-related death in patients with thoracic tumors receiving radiation therapy. According to Gagliardi et al. [11], adverse effects of radiation therapy on the heart are usually related to the radiation dose volume, but significant variations have been observed in the dose-volume indices proposed by various researchers, and there is no unified standard to date. This issue primarily stems from the fact that the dose-volume indices obtained from 3DCT simulation positioning under voluntary breathing disregard the influence of breathing and heartbeats, leading to substantial calculation errors for the dose-volume indices. Respiratory and cardiac motion can displace the heart, which can cause a decline in target coverage or an elevation of the dose on normal tissues, ultimately increasing the risk of treatment failure or complications [12–14]. Motion dose-volume index assessment research of the heart and its substructures is currently in its infancy.

Tong et al. [15] utilized calm inspiration electrocardiogram-gated 4D-CT to investigate volume variations in the heart, pericardium, and LVM during relaxation. The findings revealed volume variations of 16.49 ± 3.85%, 12.62 ± 3.94%, and 24.23 ± 11.35% in response to heartbeats. These findings are similar to the results of this study, in which the volume variations of the heart, pericardium, and LVM during calm inspiration at end-exhalation were 17.79 ± 3.79%, 13.45 ± 3.33%, and 28.14 ± 8.39%, respectively. Johnson et al. [16] examined 15 patients with coronary heart disease using digital subtraction X-ray angiography. The study found that the left anterior descending and left circumflex coronary arteries exhibited significant motion in the head-foot direction relative to the LR and front-back directions, which is comparable with the results of the present study. Li et al. [17] identified larger motion amplitudes in the AP and head-foot directions of the AS than those of the VS in their AS study. This finding aligns with the observed motion patterns of the VS and AS in response to heartbeats during the three breathing states evaluated in this research.

This study revealed a fundamental similarity in the volume and shape variations of the heart, pericardium, LV, RV, and RVM across all breathing states. Similarly, the volume and shape variations of the LVM and AS also displayed a basic consistency. However, the AS exhibited a distinct pattern of changes relative to the heart and pericardium. These findings suggest that relying on conventional dose-volume indices for the heart or pericardium to represent cardiac substructures can result in substantial errors.

The comparison of heart and substructure volumes revealed basic consistency in the volume and shape change patterns between EIBH and EEBH. The primary dissimilarity lies in the LR motion of the VS and the front-back motion of the RV. However, for patients with esophageal cancer or central lung cancer undergoing radiation dose evaluation, the effects of LR and front-back direction movements must be considered. Conversely, for breast cancer patients undergoing radiation therapy, increased front-back direction movements can cause an increase in the volume entering or approaching the irradiation range, resulting in a potentially significant dose escalation.

Compared with EIBH, DIBH led to a significant reduction in the volume of the heart and its substructures—except for the LVM—as well as a notable decrease in LR displacement of the heart, LV, RV, RVM, and VS. This effect primarily results from heightened thoracic pressure during DIBH, which causes compression of the heart and its substructures, resulting in decreased volume and LR displacement. Simultaneously, there is a significant reduction in the magnitude of the front-back motion of the LV, RVM, and AS, which can limit the entry of the heart and its substructures into the radiation range for therapies for thoracic wall tumors, breast cancer, and other cancers.

Observations of displacement changes in the heart and substructures during the three breathing states attest to the highly irregular motion present throughout the cardiac cycle. The most noteworthy motions of the heart, pericardium, and pLCX occur in the head-foot direction, whereas the LR direction displacement of the RV and RVM is greatest, and the front-back motion is the most pronounced for the LV and LVM. The motion patterns of the pmLAD and AS are similar in both the front-back and head-foot directions. The displacement of the VS is similar across all three directions.

The HD_MAX_ of the heart and its substructures are both greater than 11 mm. HD is very sensitive to abnormal changes. Breath holding at the end of deep inspiration can reduce the HD_MAX_ of cardiac substructures except for the pericardium and left ventricular cavity. The displacement and deformation of cardiac substructures vary in each direction. Overall, the deformation in the apical direction is greater than that in the base direction. The changes in the ventricles and myocardium are relatively large and are mainly related to heart function.

The participants in this study comprised a healthy population. We employed magnetic resonance imaging, a radiation-free scanning method that requires multiple repeated breath holdings over a lengthy duration, which demands a high level of physical fitness. Young and healthy participants are able to better control respiratory repeatability in different states, resulting in higher-quality MR images. By obtaining healthy cardiac images, a cardiac motion model was developed, which provides a reliable basis for analyzing the assessment errors in cardiac doses. In future studies, we plan to apply the established motion model of the heart and its substructures in different breathing states to patients with thoracic tumors, such as breast cancer, esophageal cancer, and lung cancer. Our goal is to examine the differences in dose-volume indices caused by different breathing states and to facilitate practical clinical applications.

## Conclusion

Our quantitative analysis of the effects of cardiac motion during three breathing states on the motion state of the heart and its substructures indicated significant differences in the displacement and shape changes of these structures. When evaluating radiation doses for thoracic tumor radiotherapy, it is essential to consider the combined effects of cardiac motion and breathing on the heart and its substructures when evaluating dose-volume indices.

## Data Availability

Not applicable.

## Abbreviations

4DMR: Four-dimensional magnetic resonance
pLCX: The proximal portion of the left circumflex coronary branch
AP: Anterior–posterior
AS: Atrial septum
EEBH: End-expiration breath holding
EIBH: End-inspiration breath holding
DIBH: Deep end-inspiration breath holding
DSC: Dice similarity coefficient
ROI: Region of interest
RV: Right ventricle
RVM: Right ventricular myocardium
LR: Left–right
LV: Left ventricle
LVM: Left ventricular myocardium
pmLAD: Proximal and middle portions of the left anterior descending branch
VS: Ventricular septum
